# Laboratory Assay of Brood Care for Quantitative Analyses of Individual Differences in Honey Bee (*Apis mellifera*) Affiliative Behavior

**DOI:** 10.1371/journal.pone.0143183

**Published:** 2015-11-16

**Authors:** Hagai Y. Shpigler, Gene E. Robinson

**Affiliations:** 1 Carl R. Woese Institute for Genomic Biology, University of Illinois at Urbana-Champaign, Urbana, Illinois, United State of America; 2 Department of Entomology and Neuroscience Program, University of Illinois at Urbana-Champaign, Urbana, Illinois, United State of America; Monash University, AUSTRALIA

## Abstract

Care of offspring is a form of affiliative behavior that is fundamental to studies of animal social behavior. Insects do not figure prominently in this topic because *Drosophila melanogaster* and other traditional models show little if any paternal or maternal care. However, the eusocial honey bee exhibits cooperative brood care with larvae receiving intense and continuous care from their adult sisters, but this behavior has not been well studied because a robust quantitative assay does not exist. We present a new laboratory assay that enables quantification of group or individual honey bee brood “nursing behavior” toward a queen larva. In addition to validating the assay, we used it to examine the influence of the age of the larva and the genetic background of the adult bees on nursing performance. This new assay also can be used in the future for mechanistic analyses of eusociality and comparative analyses of affilative behavior with other animals.

## Introduction

The care of offspring is a fundamental component of social behavior. The most widespread forms of this behavior involve maternal and paternal behaviors that contribute to the defense and sustenance of offspring who would otherwise perish. Offspring care has been well studied from both proximate and ultimate perspectives in vertebrates, especially birds and rodents, and is one of the best understood forms of affiliative behavior [1]. However, insects do not figure prominently in this topic because *Drosophila melanogaster* and other traditional models show little if any paternal or maternal care and species that do perform parental care like carrion beetles [2] and earwigs [3] still lack advanced molecular and genomic resources.

Offspring care is central to systems of eusociality, but it is usually provided cooperatively by older siblings, rather than by a parent. As in vertebrate parental care, sibling care involves high frequencies of interaction and more-or-less continuous “progressive” feeding. Cooperative brood care is an evolutionary novelty, with almost no equivalent in non-eusocial insect species, and it forms one of the three defining characteristics of eusociality [4, 5]. In complex insect societies, the queens are highly specialized for egg laying and provide no maternal care after oviposition. Worker brood care is thus a prime target for broad comparative studies of the mechanisms and evolution of affiliative behavior, but its investigative potential is hindered by the difficulty of studying this within-nest behavior. Other behaviors like aggression and foraging, which have been studied extensively in the honey bee, have been the subjects of broad comparative mechanistic analyses with other species like mouse, stickleback fish, paper wasp, and the fruit fly [6–8]. Laboratory assays of brood care behavior have been developed for some ant species, especially species studied almost exclusively in laboratory enclosures [9, 10].

Brood care is the major within-nest task performed by worker honey bees during their first two weeks of adult life, prior to the onset of foraging. Honey bee nursing behavior has been studied from diverse perspectives, including chemical communication between adult nurse bees and the brood they care for [11–13]; kin recognition during the rearing of a replacement queen due to the sudden death or disappearance of the resident queen [14–17]; the ability to differentiate between male and female larvae [18]; and the behavioral and brain transcriptomic responses of workers to brood pheromone [19, 20]. Despite this important body of work, relatively little is known about quantitative aspects of honey bee nursing behavior. Observations made with glass-walled observation hives have revealed that larvae are fed cooperatively by many workers; each larva is visited thousands of times and apparently fed hundreds of times during its five-day period of larval development, and each individual nurse bee is estimated to rear the equivalent of 2–3 younger sisters [21], but these estimates are based in part on inference, as it is not possible from observation hive studies to know precisely what transpired during a given worker visit to a cell in a honeycomb containing a larva.

Our relatively limited knowledge of honey bee brood care behavior contrasts with extensive information available for honey bee foraging [22–24] and aggression [25–27] two other cooperative tasks requiring the collective efforts of many workers. This is because foraging and aggressive behaviors occur outside and can be observed under natural conditions; this is much more difficult for behaviors that occur within the beehive. In addition, much has been learned about mechanisms underlying foraging behavior by using the proboscis extension laboratory assay [28–30]. Similarly, much has been learned about the mechanisms of aggressive behavior of individual bees and how individual behavior relates to colony defense thanks to a laboratory assay of aggression first developed by Breed [31], and then modified over the years [7, 14, 26, 32]. The assay involves measuring the response of small groups of bees to an intruder using a carefully developed index of aggression, and has been used to learn about endocrine, neurochemical, and molecular mechanisms underlying the aggressive response [7, 33–35]. By contrast, there has been no convenient assay to study honey bee nursing behavior in the laboratory.

The paucity of quantitative assays of brood care is a result of the biological realities of this behavior. Brood care takes place inside the dark hive and can only be observed without disturbance in a limited way. Moreover, because bees collectively feed each individual larva, it is difficult to quantify the performance of individual nurses. As a consequence, there is no method to quantify the intensity of nursing behavior. Either a method for direct tracking of nursing in the hive or a laboratory assay comparable to the assay for aggression would provide a platform for discovery. We developed a new laboratory assay that enables us to track the nursing behavior of individual bees as they function in small groups. We also developed several measurements for quantifying nursing behavior. In this paper, we report on the development of the method and the knowledge we obtained from it on some of the internal and external factors that influence brood care. We also measure for the first time the direct influence of single workers on the rearing of individual queen honey bees. This new method can be used also for comparative studies of affilative behavior in other species.

## Material and Methods

### General procedures

One-day-old adult bees were obtained from apiaries maintained by the University of Illinois Bee Research Facility, Urbana, IL, June–August 2014. Frames of honeycomb containing pupae were removed from colonies headed by naturally mated queens and placed in an incubator (34°±1°C, 45%±10% RH). Newly emerged adult bees (1–18 h old) were placed in groups of 10, each bee marked with a unique color for individual recognition. Bees were kept in vertically oriented Petri dishes (100 X 20 mm) with a beeswax foundation sheet placed on the base (“wall”) of the dish to mimic in-hive conditions. Dishes were supplied with one tube of honey (~1.4 ml), 30% sucrose solution (2ml) and a mixture of fresh frozen pollen and 30% sugar solution (~10 mm diameter ball). Plate diagram can be found at S1 Fig.

A group of queen larvae were raised in a queenless colony via standard commercial methods (Laidlaw and Page 1997). The queen larvae were each reared in a commercial plastic queen rearing cup (JZBZ, Location, cat #: 440). Larvae placed in such a cup are often reared as queens by workers under the right social and nutritional conditions, i.e., if they are without a queen and possess sufficient nutritional reserves. In that case they build a wax “cell” with the rearing cup as a base, and add copious amounts of royal jelly to it to promote queen development [36]. These are called “queen cells”. Four-day-old queen larvae were chosen for this assay because larvae at this age are fed for only one more day prior to the start of pupation, allowing for a short-term behavioral assay whose results can be evaluated robustly by queen survival.

The groups of workers in petri dishes were held in a walk-in incubator room (34°±1°C, 45%±10% RH) for seven days. Observations took place in the incubator room where the bees were held. On day seven, one four-day-old queen larva in a queen cell was introduced to the group through a hole in the top of the petri dish. After a queen cell was introduced to the groups, detailed observations were performed on each group for 5 min and each interaction of the bees with the cell was recorded. Preliminary observations revealed two types of behavioral interactions that the adult bees had with the larvae: short visits 2–10 sec, and long visits 11–90 sec in duration. Short visits usually included entry of only the head and the thorax into the cell, while long visits were defined by entry of the whole body and contractions of the abdomen. We interpret the short visit as “cell inspection” and long visit as “nursing,” i.e., the care and feeding of the larva, in concordance with previous reports [37, 38] and queen larvae [16, 39]. The incubator room was kept in darkness except for during observations, which were performed under typical room fluorescent lights. The petri dishes of bees were placed on observation tables for over 30 min before the beginning of the experiment and were not interrupted except for the introduction of the queen cells (one per petri dish). A video record of the assay can be found at S1 Video.

In all experiments the same queen cells were used in more than one group. The effects of temperature and humidity was assessed by conducting some assays either at 28°±1°C, 30%±5%RH or 34°±0.5 C, 45%±10% RH which is the normal microclimate inside the beehive. The behavioral response was more robust under the latter conditions (90% vs 60% of the groups showed nursing behavior, respectively; N = 20), so all subsequent experiments were conducted under those conditions.

### Experiments

#### Experiment 1: Worker response to a queen cell with a larva vs. an empty cell

This experiment was performed to determine whether the bees showed a specific response to the larva. Queen cells were introduced to groups of ten bees (N = 9) from one source colony for five minutes. We recorded the total number of times bees entered the cell (“visits”), the total time spent in the cell by all bees, and the number of bees visiting the cell in each group. As a negative control, an empty queen cup that came from the same source colony was introduced into the same groups. Cells with queen larvae or empty control cells were introduced sequentially in random order, with a 10-min interval between each test. The behavior of the bees was recorded and compared using a paired t-test. The experiment was repeated with a second colony.

#### Experiment 2: Worker response to a queen cell with a larva vs. royal jelly

This experiment was performed to determine whether the bees were attracted to the queen cells to scavenge the royal jelly in the cell rather than to provide care to the larva. Four-day-old queen cells were removed from a queenless colony. Half of the cells (N = 34) were treated as follows: the larva was removed from the cell, the royal jelly was removed, the cell was washed using sterile water, and the larva was placed back into the washed cell. In the other half, the larvae were removed, leaving only the royal jelly. Both types were introduced sequentially in random order to groups from two source colonies (N = 21) with a 30-min interval between the tests. The same measurements as above were taken and compared using a paired t-test. In addition, the cells were left in the dishes (one per dish) overnight to determine the proportion of each type that was “capped” (bees add wax to completely surround the developing queen) and successfully maintained to produce a mature queen. The proportion of cell capping in each group was compared using Fisher’s exact test for independence.

#### Experiment 3: Effect of group size on nursing performance

This experiment quantified the success of brood care in this laboratory assay by determining the effect of group size on brood care and the minimum number of bees needed for successful growth of the larva. Queen cells with four-day-old larvae were introduced to experimental groups (from three source colonies) of three different sizes: eight bees (N = 65), one bee (N = 38) or zero bees (N = 24, an empty dish), and were left in the dish for ten days. The honey bee queen larva goes into the pre-pupa stage after it spins a cocoon at age of five days, while the workers seal the cell with wax (Laidlaw and Page 1997). Each larva was monitored to see whether it spun a cocoon and had its cell capped with beeswax by the workers, died inside the cell, or died after falling out of the cup onto the dish floor during the first 24 h period (larvae given to zero bees could not be capped, but still could spun a cocoon). Adult queen eclosion out of cells contain cocoon or capped was monitored 7–9 days later. We compared the proportion of cells contain cocoon or capped relative to the total number introduced; the proportion of queens that successfully eclosed relative to the total number of introduced cell and relative to the number of cells contain cocoon or capped; using Fisher’s exact tests. Fisher’s exact test with Bonferroni correction was used for pairwise comparisons. The three source colonies used to make the groups were sampled equally.

#### Experiment 4: Effect of larval age on nursing behavior

The effect of larval age on nursing behavior was evaluated by giving worker groups either 3-day-old or 4-day-old queen larvae reared and removed from the a queenless colony as detailed above. Queen larvae were reared as detailed above and were removed from the queenless colony when they were 3 or 4 days old. The larvae were introduced to groups of ten 7-day-old bees from single source colony (N = 10, for each experimental group) and detailed observations were made as in Experiment 1. The results were assessed using a two-tailed student t-test.

#### Experiment 5: Effect of adult worker genetic background on nursing behavior

This experiment determined whether the assay is capable of detecting genetic differences in brood care behavior by comparing the performance of bees from different genetic sources. Genetic effects on nursing behavior have been documented in studies performed with whole colonies in observation hives [17]. We compared nursing behavior in bees derived from three different unrelated queens (N = Colony A: 65; B: 59; C: 54), each instrumentally inseminated with semen from a single, unrelated drone (male). The latency to first visit, number of nursing visits, number of inspections, total time in the cell and number of visiting bees were compared across the colonies using one-way ANOVA and LSD post-hoc tests. The proportion of groups interacting with the queen cell from each source colony was compared using Fisher’s exact tests for independence.

### Statistical analyses

All statistical analyses were performed using SPSS 22.0 software (IBM). Observations for Experiments 1–4 were performed solely by the first author, and observations for Experiment 5 were performed by the first author and two additional observers, each one performing observations on groups from all three source colonies. No significant differences were found between the observers (One way ANOVA P > 0.1, for all measurements; N > 120)

## Results

### Experiment 1: Worker response to a queen cell with a larva vs. an empty cell

**Fig 1:**
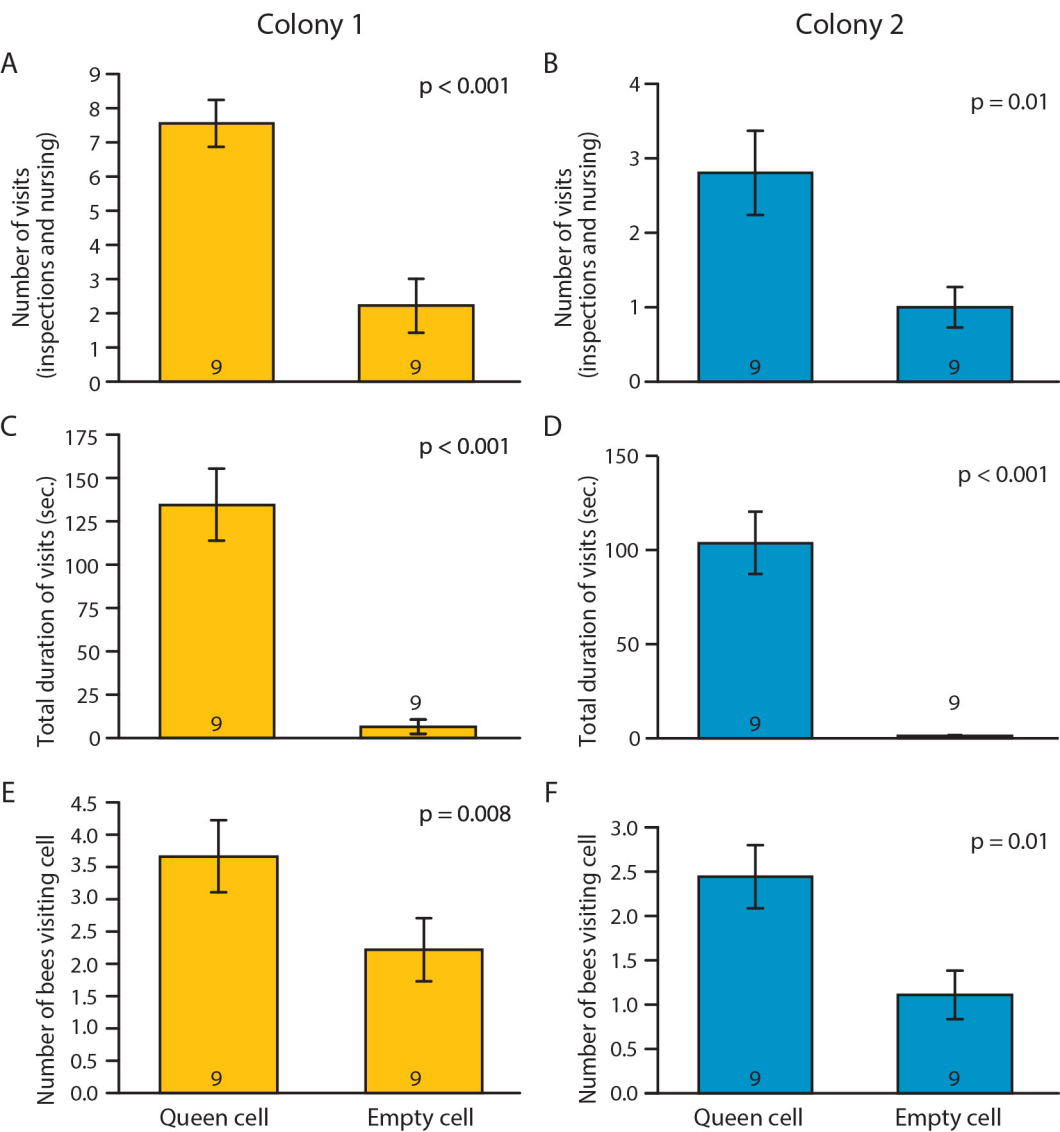
Worker response to a queen cell with a larva vs. an empty cell. Number of visits (inspections and nursing, A and B), total time spent visiting (C and D) and number of bees visiting cell (E and F), for bees from Colony 1 (A, C, E) and Colony 2 (B, D, F). Data represent average ± SE, sample size (number of groups) is at the base of each bar, p-values summarize the results of paired t-test (A: *t*_*(8)*_ = 6.4; B: *t*_*(8)*_ = 7.1; C: *t*_*(8)*_ = 3.4; D: *t*_*(9)*_ = 3.0; E: *t*_*(9)*_ = 5.3; F: *t*_*(9)*_ = 3.1).

Bees made significantly more visits and spent significantly more time in the queen cells that contained larvae compared with empty queen cells (Fig 1). The number of bees that responded to a queen cell with a larva also was significantly higher than toward an empty queen cell. Results were consistent for bees from two different unrelated colonies (Fig 1).

### Experiment 2: Worker response to a queen cell with a larva vs. royal jelly

**Fig 2:**
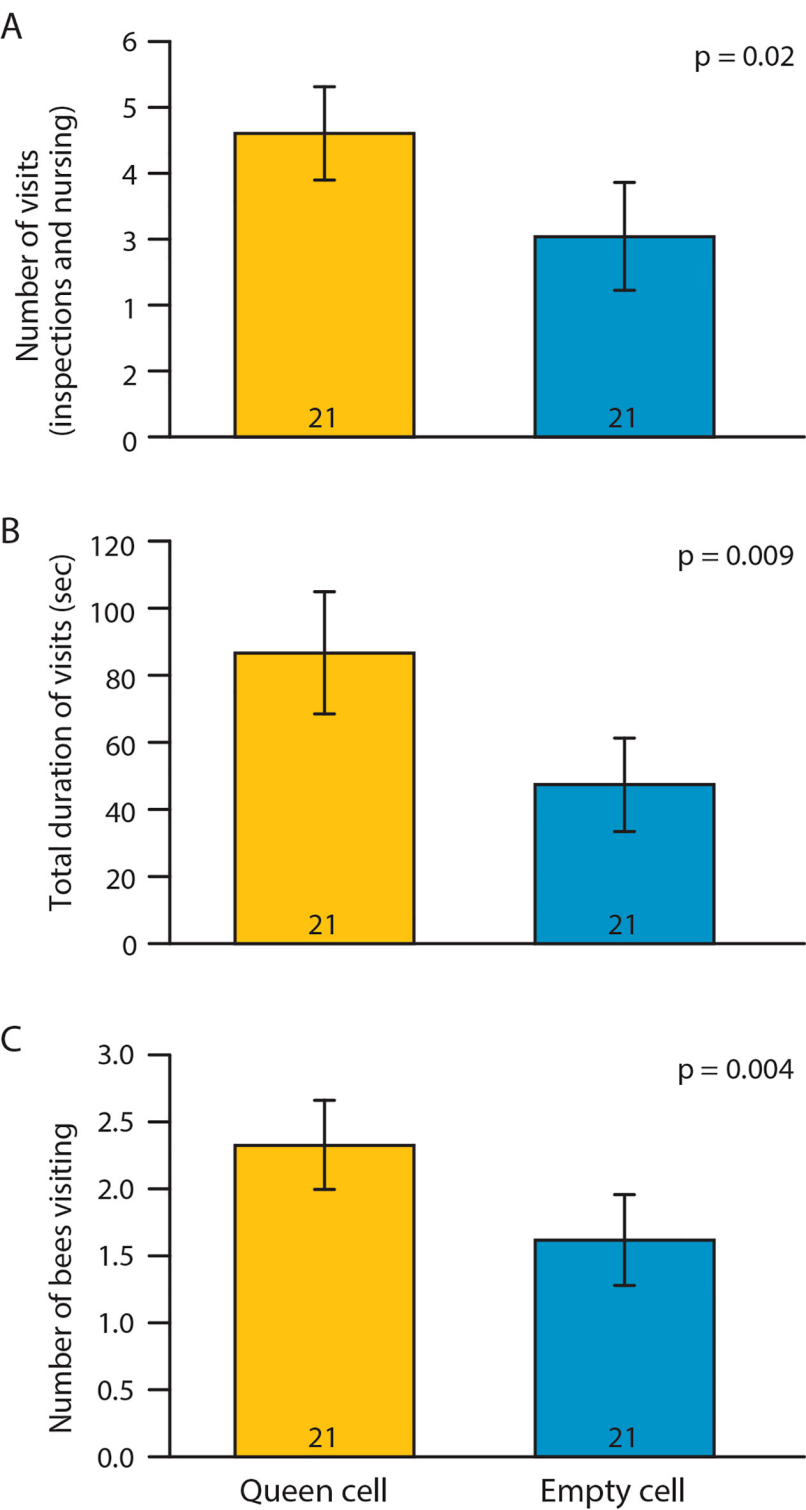
Worker response to a queen cell with only larva vs. a queen cell with royal jelly. Number of visits (A), total time spent visiting (B) and number of bees visiting cell (C). Scan sampling of 21 groups (10 bees in each group) during the first 5 minutes after the introduction of either a queen larva or royal jelly. Data represent average ± SE, sample size (number of groups) is at the base of each bar, p-values summarize the results of paired t-test (A: *t*_*(20)*_ = 2.5; B: *t*_*(20)*_ = 2.9; C: *t*_*(20)*_ = 3.25).

Bees made significantly more visits and spent more time in queen cells with a larva compared with those with royal jelly (Fig 2). The number of bees that responded to cells with queen larvae also was significantly higher than to cells with royal jelly. Bees capped 9 out of 17 queen larva cells but none of the 17 cells with only royal jelly (Fisher's exact test p = 0.001). None of the capped larvae developed into mature queens, presumably because our removal of royal jelly to set the experiment up left them with an insufficient reserve that could not be replaced by the bees in the group.

### Experiment 3: Effect of group size on nursing performance

**Fig 3:**
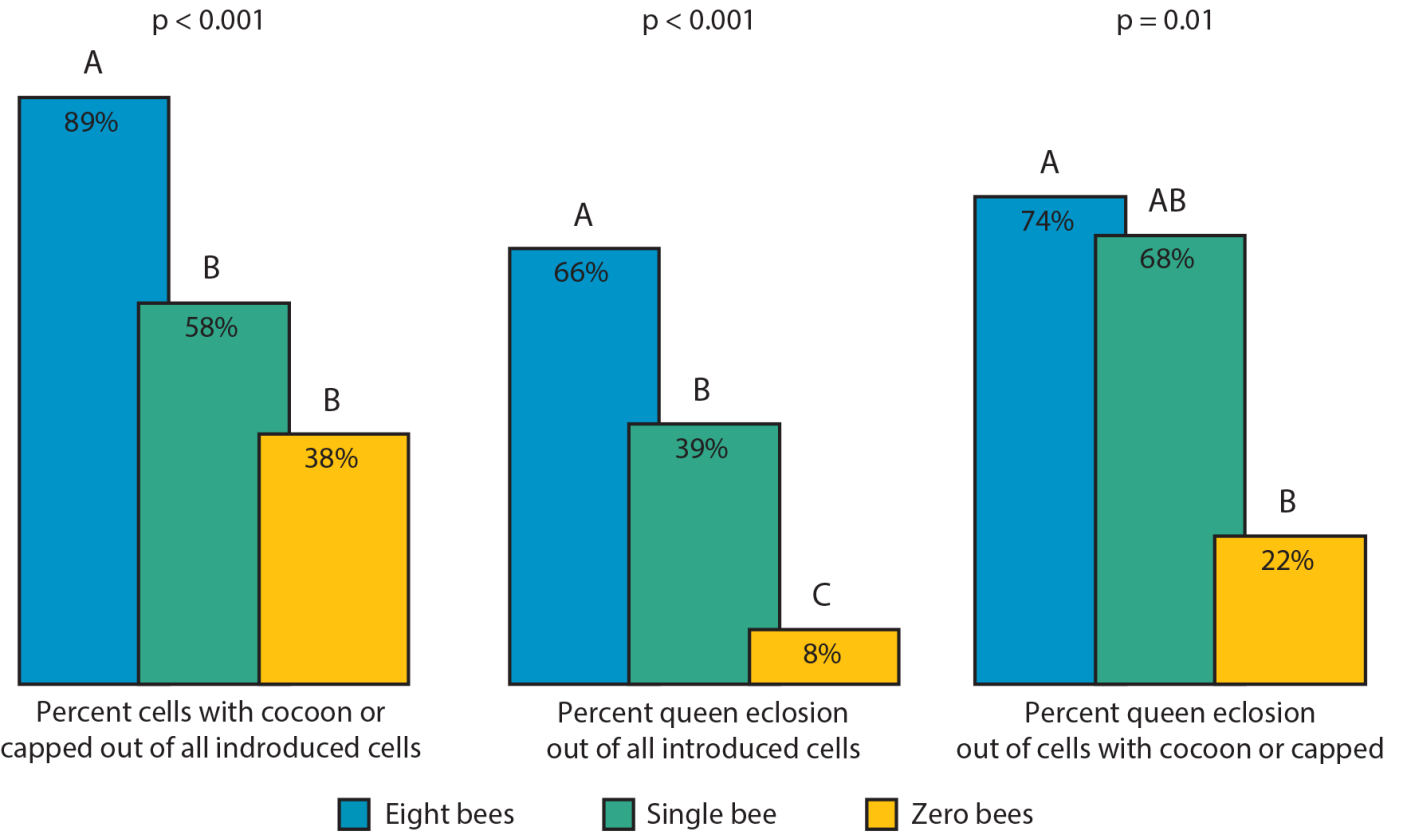
Effect of group size on nursing performances. Percentage of: cells with cocoons or capped / total number of queen cells tested (left); queens that eclosed / total number of queen cells (middle); and queens that eclosed / cells with cocoon or capped (right) in three experimental groups: 8-bees (blue; N = 65), single-bee (green; N = 38) and zero-bees (yellow; N = 24). The p-values summarize the results of Fisher’s exact test for independence, different letters in parentheses indicate a significant difference in proportion between the groups in a pairwise test (Fisher's exact test with Bonferroni correction for multiple testing p < 0.05).

Nursing performance was assessed by quantifying the proportion of cells contain a pupae in a cocoon or capped by the workers, the proportion of adult queens that eclosed /all cells, or the proportion of queens that eclosed / cells containing a pupae in a cocoon or capped by the workers, when cared for in groups of eight bees, one bee or zero bees. The cocoon / capped counts are complementary to the percentage of larvae that died during the first 24h. There were group size effects: the proportions of all three measurements were significantly different from random distributions (Fisher’s exact test, p ≤ 0.01) (Fig 3). Queen larvae fared significantly better in all three measures when placed in 8-bee groups compared with dishes with zero adult bees. Eight-bee groups also had higher proportions of pupae with cocoons, pupae in cells that were capped and queens eclosing compared with one-bee-groups. Surprisingly, the proportions of queens eclosing from cells containing pupae with cocoons or of queens eclosing from cells containing pupae in cells that were capped were similar for the eight-and one-bee groups (Fisher's exact test, p = 0.78). The proportion of queens that eclosed was higher in the one-bee group than the zero-bee groups. However, one-bee groups did not show increased proportions of cells containing pupae with cocoons or pupae in cells that were capped relative to the total number of cells (p = 0.19) compared with the zero-bee experimental group. This was also the case for queens that eclosed relative to the cells containing pupae with coccoons or capped cells (p = 0.12) (Fig 3). These results maybe a result of low statistical power.

### Experiment 4: Effect of larval age on nursing behavior

**Fig 4:**
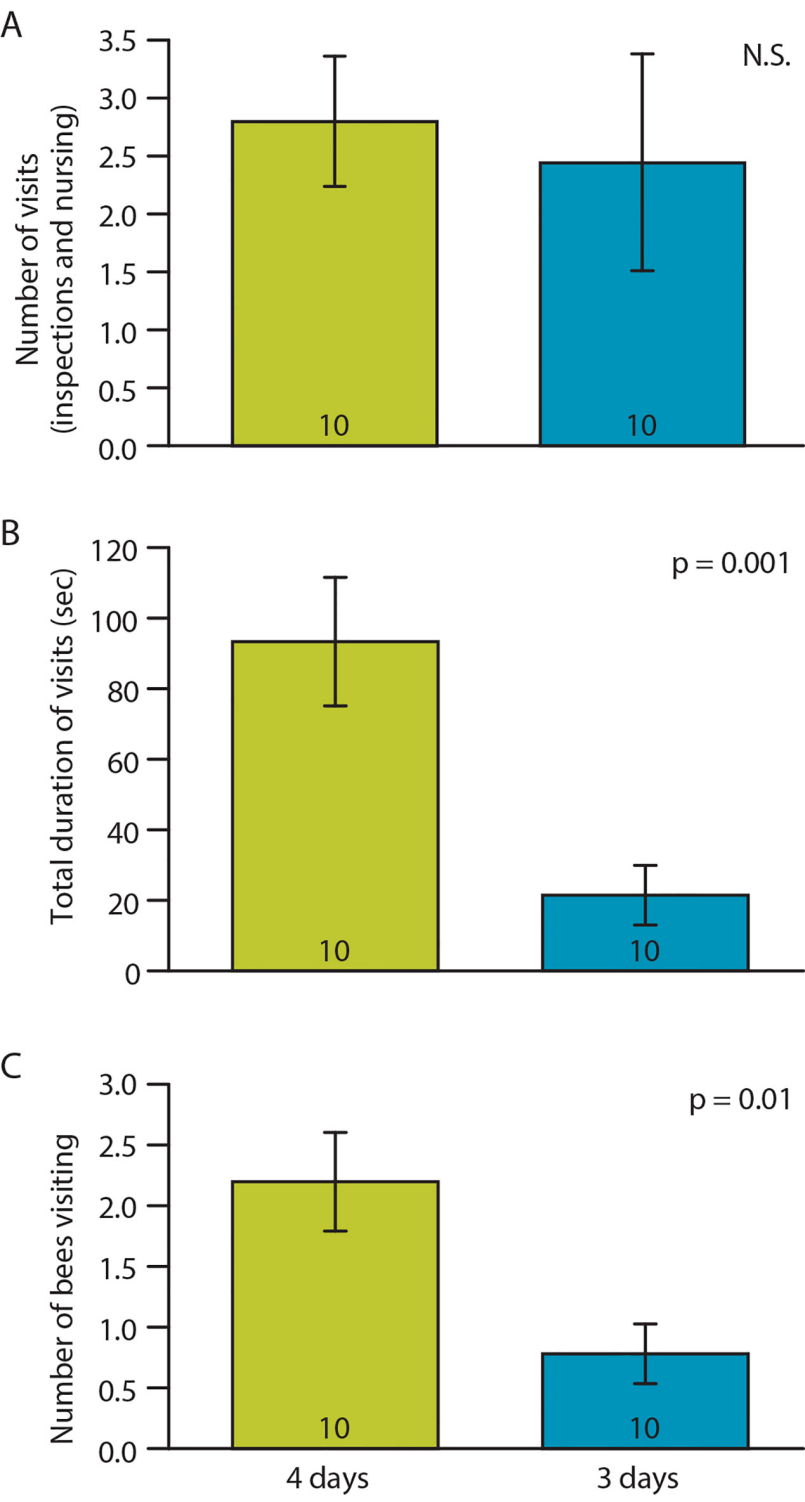
Effect of larval age on nursing behavior. Number of visits (A), total time spent visiting (B) and number of bees visiting cell (C). Data represent average ± SE, sample size (number of groups) is in the base of each bar, p-values summarize the results of two-tailed t-test (A: *t*_*(18)*_ = 0.1, B: *t*_*(18)*_ = 3.75, C: *t*_*(18)*_ = 2.86).

The number of visits made by worker bees to a queen larva was similar regardless of larval age (Fig 4). However, visit duration was significantly longer for 4-day-old compared to 3-day-old larvae. This is consistent with the fact that 4-day-old larvae are larger and require more feeding [40]. In addition, the number of bees performing visits was higher for 4-day-old compared to 3-day-old larvae.

### Experiment 5: Effect of adult worker genetic background on nursing behavior

**Fig 5:**
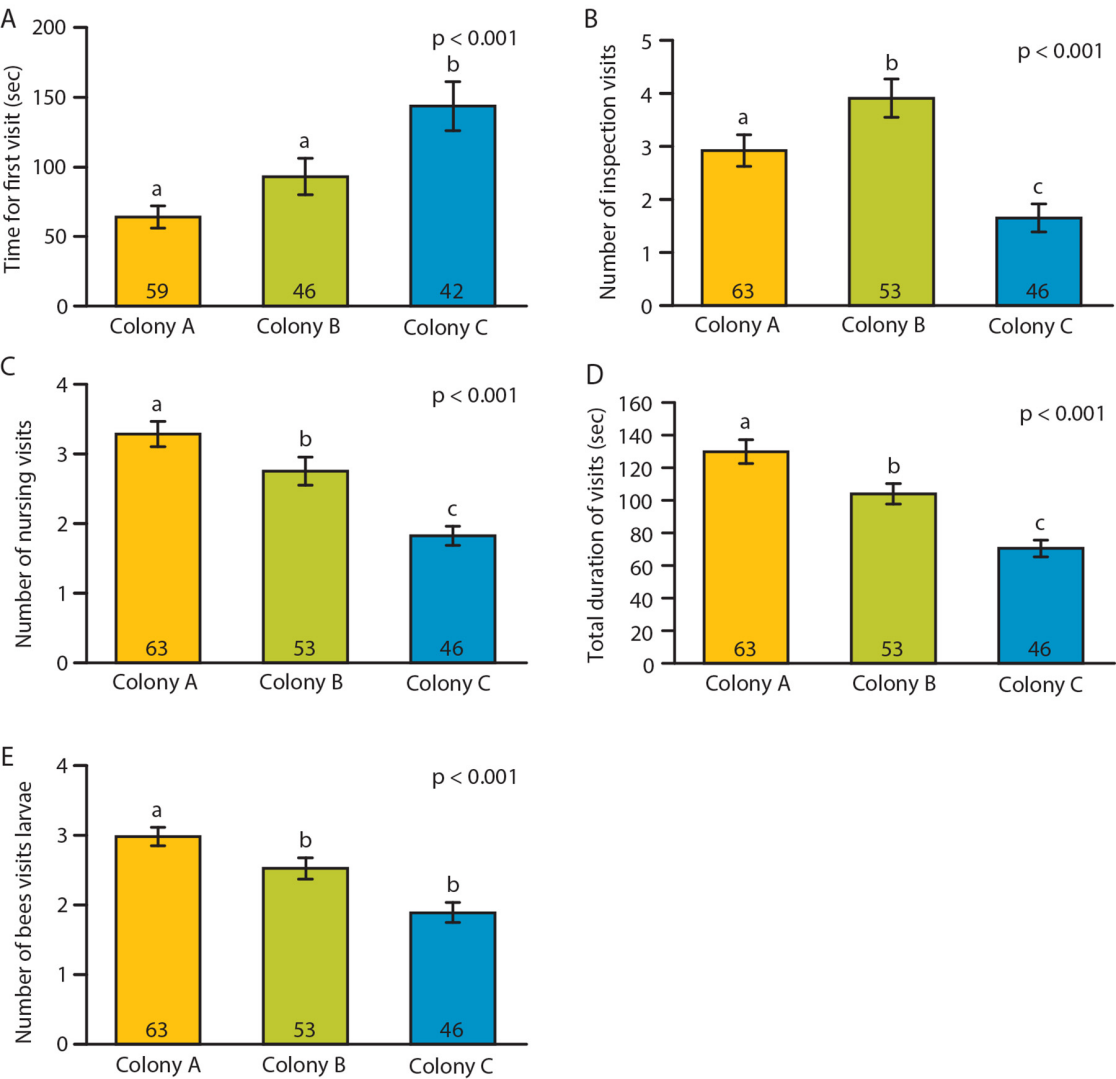
Effect of adult worker genetic background on nursing behavior. Bees from three genetically different colonies were studied. Latency until first cell visit (A); total number of inspection visits (B); total number of nursing visits (C); total time spent visiting larvae (D); and number of bees visiting cell (E). The p-value summarizes the results of one-way ANOVA (F_(2)_ ≥ 10.4 for all measurements), different letter above the bars indicate significant pair-wise differences in LSD post-hoc test (p < 0.05).

The percentage of groups performing nursing behavior was 97% (N = 65), 90% (N = 59) and 85% (N = 54) for colonies A, B and C, respectively (Fisher’s exact test, p = 0.07). In addition to the three behavioral measures used in the previous experiments, we also measured the latency to first interaction between the workers and the larva. Also due to the large sample size, we had enough statistical power to divide the visits into inspection and nursing visits and compared each one separately. We found significant effects of genetic background in all five measurements (Fig 5).

## Discussion

We present a new laboratory behavioral assay to study honey bee nursing behavior. The assay avoids the problems of working with whole colonies, is conducted under normal light conditions, and causes minimal interruption to the bees. Our results demonstrate that the assay is specific for nursing behavior and allows detailed quantification and comparison across biologically relevant groups. This assay also will be useful for cross-species analyses of affiliative behavior, since it is highly sensitive to external and internal factors.

Results presented here demonstrate the specificity of the assay. Bees showed nursing behavior in over 85% of the trials, indicating high responsiveness to the queen larva. In addition, they were much more attracted to queen cells than to empty cells. This finding suggests that the visits of the worker bees are not an exploration of a new object, but rather represent a specific response to the queen larva. This conclusion is supported by findings that bees were significantly more attracted to a queen larva than to royal jelly, even when the queen larva was in a cell without royal jelly. This result also eliminates the possibility that the bees’ attraction to the queen cell was based on food scavenging rather than nursing. We also found that the bees capped only cells with larvae and not cells containing only royal jelly, which represents a specific behavior in response to signals coming from the larva and not from the royal jelly.

Another indication of assay specificity was the increased nursing behavior displayed towards 4-day-old larvae. It is known that 4-day-old larvae are larger and thus require more food. Increases in visit length and the number of visiting bees suggest that the signals produced by 4-day old larva are different and more attractive to the bees than those produced by 3-day-old larvae. This is consistent with findings that workers can assess larval age via changes in the composition of brood pheromone and adjust their behavior accordingly [40].

The results reviewed above demonstrate a specific attraction of adult worker bees to the queen larvae, but are the bees effective at rearing a queen under these artificial conditions? Based on our findings, the answer is yes. A much higher proportion of adult queens that eclosed when larvae were reared by groups of bees compared with larvae placed in dishes without adult bees. This demonstrated the effects of brood care in the dish for successful development of larvae into mature queens.

Results of other experiments reported here provide insights into the variables that are important in determining the efficiency of the assay, which should be useful to other scientists contemplating its use. First, we showed that it is possible to obtain accurate and reliable measures of nursing activity with only a five-minute, focal group observation. This means the assay can be used in a highly efficient manner to screen nursing behavior in a large number of groups. Second, we developed a set of behavioral measures to precisely quantify nursing behavior and provide the means for detailed analyses of inter-individual differences. This can be very useful in future mechanistic studies. Third, we showed that larval age has an effect on the intensity of nursing behavior, as mentioned above.

The differences detected between workers derived from three different queens suggest that this assay is capable of detecting genetic variation in nursing behavior. This also is an encouraging result because genetic variation in the intensity of nursing behavior directed toward queen or worker larvae has been reported [17, 39], and inferred differences in intensity of nursing behavior have also been reported for strains of bees selected to collect high and low amounts of pollen [41]. The ability to directly measure this type of behavioral variation will be useful in elucidating the genetic and molecular bases of nursing behavior.

Observations with this assay revealed two surprising findings. First, although the presence of even one adult worker is very important in ensuring that a queen larva spins its cocoon safely without falling out of its (vertically oriented) cell; some queen larvae are able to seal themselves in cells without the assistance of adult workers. Second, single workers can nurse, cap and successfully rear a queen larva (but groups are more effective). This finding provides a direct method for measuring the behavior of individual bees, which is impossible inside the colony where brood care is performed cooperatively. This opens a wide range of opportunities for studying the mechanisms of nursing behavior to better understand the intrinsic factors that control nursing behavior including pheromones, hormones, and intracellular signaling mechanisms. The assay can also be used to explore the effects of extrinsic factors, such as parasites, pathogens, and pesticides, on nursing behavior, a topic of increasing importance given the recent large-scale losses of honey bee colonies due to Colony Collapse Disorder [42].

Another possible use of this assay is to explore the evolution of cooperative brood care in honey bees. Queens are reared on royal jelly which is placed in the queen cell while the larva is still young, and the queen cell is capped before the larva has finish eating its contents. The larva will continue to feed on royal jelly in the capped cell for about 24 hours before entering the pupal stage. By contrast, honey bee worker larvae are fed progressively by nurses and capped without any food in the cell [21]. Progressive provisioning has been recorded only in some of the social bee species and not in the solitary bee species; solitary bees, as well as some of the eusocial species like the stingless bees, rear all their offspring, worker and queen, by mass provisioning in which all the food is gathered in the cell before the egg is laid. This suggests that mass provisioning is the ancestral feeding strategy in bees, while progressive feeding evolved later in some lineages with the emergence of eusociality. Honey bee queen larva provisioning is more similar to the ancestral strategy of mass provisioning than worker provisioning, suggesting that the ancestral nursing strategy is conserved in honey bee queen rearing. Our findings that an “orphaned” larva can complete the growth cycle and that single bees engage in nursing of queen larva support this speculation.

Cooperative brood care in social insects also can be important for comparative analyses of affiliative behavior in general, especially the care of young, even though the care is provided by siblings, rather than by parents as in vertebrates. This is especially the case because the two model genetic invertebrate species, *Drosophila melanogaster* and *Caenorhabditis elegans*, do not care for their young. Honey bees can thus be employed usefully in comparative analyses because they bring the typical invertebrate advantages of short generation time and economical rearing, in addition to a growing arsenal of genomic and genetic resources [43–45]. Comparing honey bees with other insects that show parental care will be interesting in exploring the evolution of various forms of insect sociality. Comparing to mammals also will be interesting in terms of broad evolutionary analysis because there are intriguing similarities between honey bee “allomaternal” and mammalian maternal care. Both are progressive and occur across the circadian cycle [46–48], both involve glandular secretions (royal jelly and milk) rather than food collected directly from the environment, and both are performed by individuals that are not in a reproductive state (for mammals only during the period devoted to nursing; worker honey bees are almost completely non-reproductive during their entire life). The new nursing behavior assay we present here should increase the utility of honey bees in exploring the molecular foundations of this important affiliative behavior.

## Supporting Information

S1 Fig Plate diagram. Diagram of a Petri dish used in the laboratory brood care assay, the plate stands on wooden base and is positioned vertically.
(EPS)

S1 Video Video recording of the laboratory brood care assay. Each bee in the group is marked with a unique color dot for individual identification (Filmed with Canon PowerShot S5-IS, and edit with Microsoft Movie Maker).
(MP4)
